# *In silico* medical device testing of anatomically and mechanically conforming patient-specific spinal fusion cages designed by full-scale topology optimisation

**DOI:** 10.3389/fbioe.2024.1347961

**Published:** 2024-09-10

**Authors:** Thijs Smit, Niels Aage, Daniel Haschtmann, Stephen J. Ferguson, Benedikt Helgason

**Affiliations:** ^1^ Institute for Biomechanics, ETH Zürich, Zürich, Switzerland; ^2^ Solid Mechanics, Technical University of Denmark, Kongens Lyngby, Denmark; ^3^ Department of Spine Surgery and Neurosurgery, Schulthess Klinik, Zürich, Switzerland

**Keywords:** *in silico*, ASME V&V40, model credibility, medical device testing, topology optimisation, patient-specific, finite element analysis, lumbar spinal fusion implant

## Abstract

A full-scale topology optimisation formulation has been developed to automate the design of cages used in instrumented transforaminal lumbar interbody fusion. The method incorporates the mechanical response of the adjacent bone structures in the optimisation process, yielding patient-specific spinal fusion cages that both anatomically and mechanically conform to the patient, effectively mitigating subsidence risk compared to generic, off-the-shelf cages and patient-specific devices. In this study, *in silico* medical device testing on a cohort of seven patients was performed to investigate the effectiveness of the anatomically and mechanically conforming devices using titanium and PEEK implant materials. A median reduction in the subsidence risk by 89% for titanium and 94% for PEEK implant materials was demonstrated compared to an off-the-shelf implant. A median reduction of 75% was achieved for a PEEK implant material compared to an anatomically conforming implant. A credibility assessment of the computational model used to predict the subsidence risk was provided according to the ASME V&V40–2018 standard.

## Highlights

• A full-scale topology optimisation formulation to design anatomically and mechanically conforming patient-specific spinal fusion implants was tested, *in silico*, on a cohort of seven patients.
• The anatomically and mechanically conforming patient-specific spinal fusion cages reduced the median subsidence risk by 89% for titanium and 94% for PEEK implant materials compared to an off-the-shelf implant.
• The method was similarly effective for patients with low and high bone quality.
• The credibility of the *in silico* medical device testing procedure was evaluated according to the ASME V&V40–2018 standard.

## 1 Introduction

Recent advancements in patient-specific computational models enable *in silico* clinical trials to play an increasing role in the development of medical devices. The benefits of *in silico* clinical trials over conventional clinical trials include 1) cost and time efficiency; 2) collecting data before animals or humans are subjected to potential harm; 3) comparing multiple treatments per patient; and 4) a broader or more in-depth analysis of treatments (Viceconti et al., 2021); (Action, 2015).

In recent years, there has been a surge in the use of *in silico* clinical trials, e.g., in the fields of pharmacology, cardiovascular stents, diabetes treatment, and orthopaedics (Passini et al., 2017; EMA, 2018; Sarrami-Foroushani et al., 2021; Schmitzer et al., 2022; La Mattina et al., 2023). Looking specifically at *in silico* clinical trials where finite element (FE) models were used, Kassab-Bachi et al*.* studied the effect of geometric variability in the spine on the biomechanical response, i.e*.*, the intradiscal pressure and the facet joint contact pressure in a cohort of 152 patients (Kassab-Bachi et al., 2023), and Aldieri et al*.* investigated the fracture risk of the proximal human femur (Aldieri et al., 2023) with a focus on proving the credibility of the computational models according to the ASME V&V40–2018 standard (The American Society of Mechanical Engineers ASME, 2018). With further development, *in silico* clinical trials will potentially be accepted by regulatory agencies as supplementary material for market approval applications and may be used as a (partial) replacement of animal and human clinical trials (Viceconti et al., 2016) (Viceconti et al., 2005).

The authors recently developed a topology optimisation (TO) strategy that automates the design of cages used in instrumented transforaminal lumbar interbody fusion (TLIF). The strategy incorporates the mechanical response of the adjacent bone structures in the optimisation process, with the goal to reduce subsidence risk. This results in anatomically and mechanically conforming devices (AMCDs) that successfully reduce the subsidence risk compared to off-the-shelf (OTS) cages and anatomically conforming devices (ACDs) (Smit et al., 2024) (Smit, 2023). The optimisation and *in silico* testing process have been automated further to facilitate *in silico* medical device testing on multiple patients. Furthermore, the optimization and testing procedures were modified with the goal to increase the effectiveness of optimisation and improve the credibility of the *in silico* testing process. As Viceconti et al*.* suggested, the evaluation of the credibility of the computational model should be considered early in the development of new *in silico* methods using guidelines from regulatory agencies and examples e.g. Viceconti et al. (2021).

Thus, the aim of this study was to perform *in silico* medical device testing of AMCDs on a cohort of seven patients and compare the AMCDs to OTS cages and ACDs, using titanium and PEEK implant materials. The credibility assessment of the computer model that was used in the subsidence risk prediction was carried out according to the ASME V&V40–2018 standard. We hypothesized that AMCDs reduce the subsidence risk over ACDs and OTS cages for patients with a broad range of bone quality. Furthermore, we hypothesised that PEEK cages have lower subsidence risk than titanium cages and that there is a negative correlation between the subsidence risk and bone quality.

## 2 Methods

### 2.1 Topology optimisation

**FIGURE 1:**
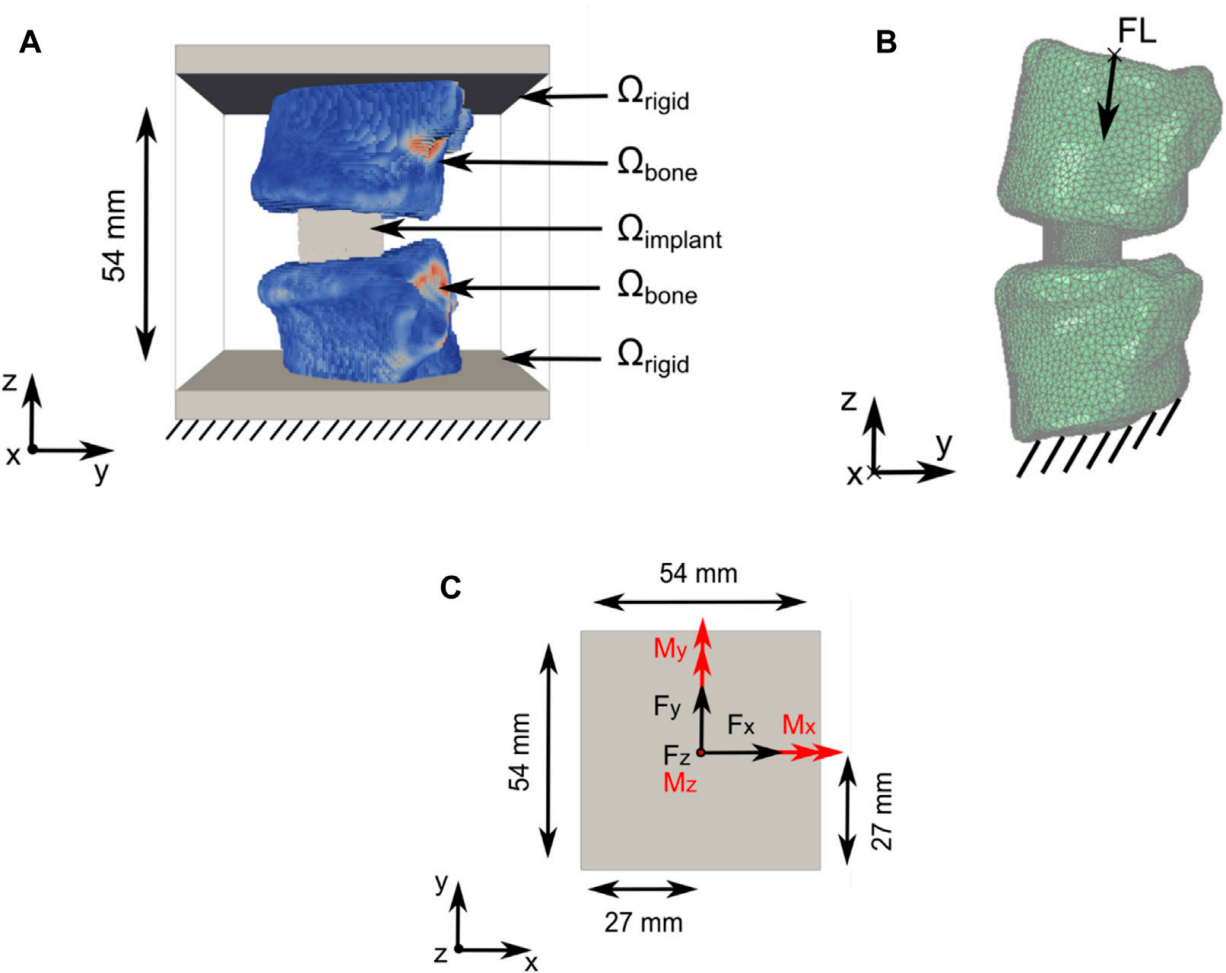
Computational domain and load application for the TO and subsidence risk assessment domain. **(A)** Optimisation domain Ω with Ω = Ω_implant_ U Ω_bone_ U Ω_rigid_. Support constraints, in all directions, were applied to the bottom of Ω (data from patient 2; see Section 2.2) **(B)** Subsidence risk assessment domain with FL application and support constraint illustrated including the right-handed orthogonal coordinate system (data from patient 1; see Section 2.2). **(C)** Forces in black and moments in red were applied on top of the optimisation domain Ω in the xy-centre of Ω_rigid_.

Our newly developed TO strategy (Smit et al., 2024) (Smit, 2023) to optimise spinal fusion cages was, in this work, slightly modified to improve its effectiveness and to allow the optimisation of titanium and PEEK implant materials. The computational domain Ω comprised an implant domain (Ω_implant_), a bone domain (Ω_bone_), and a rigid domain (Ω_rigid_), with Ω = Ω_implant_ ∪ Ω_bone_ ∪ Ω_rigid_ (Figure 1A). Ω_bone_ contains the bone structures and patient-specific material properties. Ω_implant_ was the design domain that was subjected to optimisation in the TO process.

Domain Ω, with a size of 54 mm × 54 mm × 54 mm, was discretised using a structured grid of 1.728 million hexahedral elements with an isotropic element edge length of 0.45 mm. Resampling was performed using SimpleITK version 2.1.1.2 (Lowekamp et al., 2013) to adjust the CT scan resolution to match the resolution of Ω and to position the Functional Spinal Unit (FSU) in the approximate centre of Ω. The vertebrae were partially embedded in Ω_rigid_.

Six load cases (axial compression, lateral shear, posterior–anterior shear, flexion, lateral bending, and axial rotation) were used in the TO process. The applied loads were based on the study by Rohlmann et al. (2008) and the OrthoLoad dataset (Bergmann and Damm, 2008) (Figure 1C; Table 1). Loads were applied on nodes at the top of Ω. Nodes on the bottom of Ω were fixed in all directions. The load magnitude for all patients was scaled with respect to the patient’s body weight and the body weight of patient 1 (see Section 2.2), as was used in the subsidence risk assessment (Han et al., 2013) (Zhang et al., 2017).

**TABLE 1:** Spinal loads during daily living applied in the TO process, derived from the study by Rohlmann et al. (2008) and the OrthoLoad dataset (Bergmann and Damm, 2008) from a patient with similar weight to patient 1 (see Section 2.2) [table from Smit et al. (2024) and Smit (2023)].

Description	Component	Patient 1	Unit
Axial compression	$F_{z}$	−177	N
Posterior/anterior shear	$F_{y}$	27	N
Lateral shear	$F_{x}$	5	N
Flexion	$M_{x}$	0.4	Nm
Lateral bending	$M_{y}$	0.5	Nm
Axial rotation	$M_{z}$	0.7	Nm

Similar to the TO formulation that was previously used (Smit et al., 2024) (Smit, 2023), the compliance of the bone–implant system was minimised for all load cases (optimisation objective), but to avoid the overloading of the adjacent vertebrae, the maximum principal strains in Ω_bone_ were constrained (optimisation constraints). Constraining the minimum principal strains was omitted because our previous work indicated that this constraint was inactive (Smit et al., 2024) (Smit, 2023) because the compression load from the implant is transferred to the shear, orthogonal to the endplate, along the periphery of the implant. In pure shear, the maximum and minimum principal strains have equal magnitudes but opposite signs, indicating that the tensile limit is reached first due to a lower threshold. Shearing is the dominant loading mode in the endplates, which was confirmed by several authors who identified shear failure of the vertebra endplates as the dominant failure mode in subsidence (Cadman et al., 2016); (Au et al., 2011). Furthermore, we use a local volume constraint on Ω_implant_ to create a porous implant structure. To promote manufacturability, the minimum strut diameter was controlled using robust TO formulation. No other manufacturing-specific constraints were added to make a comparison between titanium and PEEK fair because these materials have different manufacturability requirements.

The compliance minimisation problem with the maximum principal strain constraint and local volume constraint in discrete form is written as

$$\begin{array}{l} \min_{x}\sum_{k=1}^{lc}f{\left(x^{e}\right)}_{k}\;in\;Ω\\ \text{subject to} \end{array}$$ (1)

$$g{\left(x^{d}\right)}_{1}\leq 0.0\;in\;{Ω}_{bone},$$ (2)

$$v\left(x^{d}\right)\leq 0.0\;in\;{Ω}_{implant},$$ (3)

where lc represents the six load cases with k=1…6 , f is the objective function (Equation 1), $g_{1}$ is the constraint on the maximum principal strain response (Equation 2) for the compression load case in the Ω_bone_, and v is the local volume constraint (Equation 3) on Ω_implant_. The maximum principal strain constraint on the compression load case was used as this was the active constraint (Smit et al., 2024) (Smit, 2023) with a maximum principal strain limited to 0.73% based on the work by Bayraktar et al. (2004). The local volume constraint $v\left(x^{d}\right)$ followed the implementation published by Wu et al. (2018). The local volume constraint was applied on the dilated design $x^{d}$ , with a local volume fraction of 0.35, a filter radius of 3.5, and p-mean penalty of 16. Finally, Poisson´s ratio for Ω_implant_ was set to 0.3 for titanium and 0.43 for PEEK. The E-modulus for the titanium implant was set to 110 GPa (Niinomi and Nakai, 2011), and for the PEEK implant, the E-modulus was set to 3 GPa (Zhao et al., 2018). The optimisation was run for 24 h on a computational cluster using 1,000 cores in parallel. The final design should at least exhibit a discreteness measure that is lower than 3%. This value is considered to be an acceptable convergence to a solid/void design (Wang et al., 2011).

The resulting cage designs from the optimisation process have a voxelised surface because of voxel-based discretisation. The final designs were achieved by applying the “Iso volume” filter to the optimised cage using a contour of 0.5 as the threshold and exporting to an .stl file using vtk 9.1.0 (Schroeder et al., 1996). The resulting file was used in the subsidence risk assessment Section 2.3 and Section 2.4.

### 2.2 Study patient datasets

**FIGURE 2:**
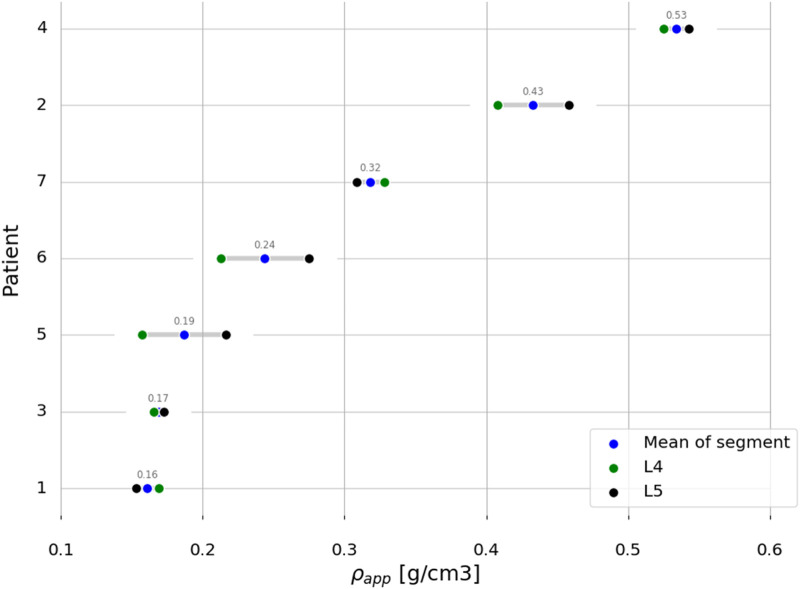
Patients ordered based on the mean segment integral vBMD.

Anonymised pre-operative computed tomography (CT) scans with clinical resolution (voxel sizes of approximately 0.30 mm × 0.30 mm × 0.50 mm), from seven female patients, were provided by the Schulthess Klinik, Zürich (Table 2). The patients, who were diagnosed with degenerative spondylolisthesis or degenerative disk disease, were treated with a TLIF procedure. Patients with other spinal diseases such as trauma, infection, or tumour were excluded. The CT images from patients 1 and 2 were reused from a previous study (Smit et al., 2024) (Smit, 2023), and all the patients provided informed consent for the use of their data for research purposes. The CT scans were calibrated using a phantom-less calibration procedure (Lee et al., 2017). The vertebrae were segmented using open-source software MITK-GEM (Pauchard et al., 2016). T-scores, based on DXA scans, were only available for the L4 level for three patients. Bone quantity was thus assessed by calculating the integral volumetric bone mineral density (vBMD) for each vertebra that was modelled, including the endplates and cortical bone in accordance with the work by Kaiser et al. (2020) (Table 2; Figure 2).

**TABLE 2:** Patient data overview. BMI, body mass index; vBMD, volumetric bone mineral density; T-scores based on DXA scans when available. NA, not available.

FSU ID	Age	Gender	BMI	Weight [kg]	Level	vBMD [g/cm^3^]	T-score
1	66	Female	18.3	51	L4	0.16	−2.3
					L5	0.15	NA
2	58	Female	25.6	75	L4	0.40	NA
					L5	0.45	NA
3	57	Female	24.2	70	L4	0.16	NA
					L5	0.17	NA
4	67	Female	41.6	100	L4	0.52	1.5
					L5	0.54	NA
5	76	Female	22.9	64	L4	0.15	NA
					L5	0.21	NA
6	76	Female	22.6	64	L4	0.21	NA
					L5	0.27	NA
7	52	Female	24.3	60	L4	0.33	1.3
					L5	0.31	NA

### 2.3 Finite element models

The model building process follows the workflow presented by Smit et al. (2024) (Smit, 2023) but is briefly described here for clarity and context. A commonly used, commercially available OTS cage, for a TLIF procedure with a height of 9 mm, was reengineered into a CAD model. The cages were placed anteriorly and across the mid-line in an optimal position, restoring spinal alignment as much as possible. The implant placement was checked by a spine surgeon according to standard surgical fusion techniques for the TLIF approach. The ACD and AMCD design domains were created by matching the top and bottom surfaces of the CAD model to the patient’s endplates. It was assumed that the intervertebral disc and cartilaginous endplates were removed during the surgery. The cages were placed according to standard surgical fusion procedures, and the implant position was checked by a spinal surgeon.

In the optimisation process (Section 2.1), daily living load cases were applied, which cause strains in the bone structures within the linear regime. The optimisation process was followed by the subsidence risk assessment (Section 2.4) that was performed in commercial FE analysis software where the cages were loaded with hyper-physiological loading that can potentially lead to non-linear material response, both in the implant and the bone structures.

**FIGURE 3:**
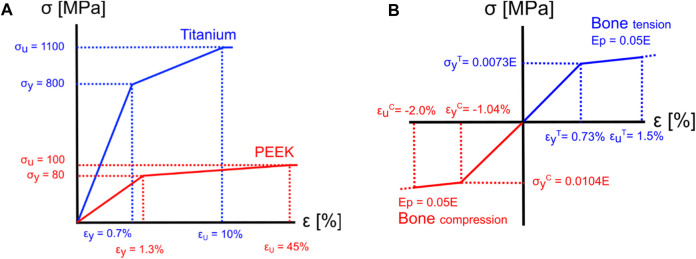
Material models for implant materials and bone. **(A)** The yield and ultimate strain were assumed to be 0.7% and 10% (Nikiel et al., 2021) for titanium and 1.3% and 45% for PEEK (Zhao et al., 2020), respectively. **(B)** For the bone elements, a tension yield strain $\varepsilon_{y}^{T}$ of 0.73% and a compressive yield strain $\varepsilon_{y}^{C}$ of −1.04% were assumed (Bayraktar et al., 2004). After reaching the yield point, a post-yield modulus (Ep) of 5% of element’s elastic Young’s modulus (E) was observed (Bayraktar et al., 2004). Subsidence risk was quantified using a maximum principal strain limit of 1.5% and a minimum principal strain limit of −2.0% (Soyka et al., 2016) (Figure is not to scale).

In the subsidence risk assessment, 10-node tetrahedral elements (C3D10M) were used to mesh the bone and implant structures with an average edge length of 1.5 mm for the vertebrae and 0.5 mm for the cages. The apparent density-to-modulus relationship from the study by Ouyang et al. (1997) (Table 5) was used to assign material properties to the bone elements in the FE mesh based on an internal calibration of the CT grey levels to the apparent density. Poisson’s ratio was set to 0.3, and the minimum value for Young’s modulus was set to 25 MPa for the bone structures. The subsidence risk assessment was performed in an explicit non-linear FE solver (Abaqus 2021, Dassault Systèmes, Vélizy-Villacoublay, France), assuming non-linear geometry and a general contact formulation. The bone structures were modelled using a rate-independent elasto–plastic material model, which captures the asymmetric tension–compression post-yield behaviour of bone (Figure 3B) (Bayraktar et al., 2004). A compressive follower load (FL) of 310 N for patient 1 was assumed based on the study by Rohlmann et al. (2014) and the OrthoLoad dataset (Bergmann and Damm, 2008). The magnitude of FL for all patients was scaled with the patient’s body weight to the body weight of patient 1 and subsequently distributed on the superior endplate of the superior vertebra such that the direction of the load was as defined by Rohlmann et al. (2009) (Figure 1B). The inferior endplate of the inferior vertebra was constrained in all translations. The models were solved on a computational cluster using 64 cores in parallel. Poisson’s ratio was set to 0.3 and 0.43 for titanium and PEEK, respectively. The material models for titanium and PEEK are shown in Figure 3A.

### 2.4 Subsidence risk assessment

The maximum factor of fracture risk (FFR) was used as a surrogate for subsidence risk. The maximum FFR is quantified as the degree of overloading of the vertebrae using a maximum principal strain limit of 1.5% and a minimum principal strain limit of −2.0% (Soyka et al., 2016). For each element “ i ” in the bone domain, the FFR ( ${FFR}_{i}$ , Equation 4) and maximum FFR (Equation 5) (Soyka et al., 2016) were calculated using

$${FFR}_{i}=\max\left(\frac{\varepsilon_{\max,i}}{1.50\%},\frac{\varepsilon_{\min,i}}{-2.00\%}\right),$$ (4)

$$Maximum\;FFR=\max\;\left({FFR}_{i}\right),$$ (5)

where $\varepsilon_{\max,i}$ is the maximum principal strain of element i , in the centroid of the element, and $\varepsilon_{\min,i}$ is the minimum principal strain of element i , in the centroid of the element.

### 2.5 Computer model credibility assessment

Credibility assessments were performed following the ASME V&V40–2018 standard. Note that the following applies to the subsidence risk assessment of Section 2.3, 2.4. The process starts with the definition of the question of interest (QoI). The next step was to formulate the context of use (CoU) including the function and scope of the computer model that was used in answering the QoI (Table 3).

**TABLE 3:** Definition of the question of interest (QoI) and the context of use (CoU).

QoI	Do the new AMCDs reduce subsidence risk compared to ACDs and OTS implants?
CoU	A patient-specific FE model (Section 2.3) will be used to quantify the subsidence risk under a hyper-physiological loading condition. The patient-specific FE models are built using clinical CT scans including the FSU of interest. The subsidence risk assessment (Section 2.4) provides an estimate, for a given patient and implant, of the factor of fracture risk (FFR). The resulting maximum FFR is used as a surrogate for subsidence risk and as an outcome variable in a comparative study. Bench tests were performed to test the implant integrity and provide validation of the FE model (Smit et al., 2024) (Smit, 2023). In a clinical setting, the surgeon would use the outcome of a patient-specific comparative study, together with other available information, his/her judgement and experience, in the treatment planning.

The model risk describes the possibility that the use of the computer model leads to a decision causing harm to the patient. The model risk is composed of the “model influence” and the “decision consequence.” The model influence describes how much influence the results of the computer model have on the decision maker.

The model influence is defined to be MEDIUM because the decision of the AMCD has lower subsidence risk than the ACD or OTS, for a specific patient, is informed by the results from the computer model, in combination with the observations and judgment of the spinal surgeon.

The decision consequence is the significance of harm to the patient in the case of an incorrect decision.

The decision consequence is defined to be LOW because a worst-case incorrect decision would lead to a patient being treated by an OTS cage, which is the current standard practise.

The selection of computer model credibility activities is based on the credibility requirements for the computational model that should correspond with the model risk that was defined (Table 4). Model credibility is defined as the trust in the predictive capability of the computer model for the CoU, according to the ASME V&V40–2018.

**TABLE 4:** Model risk summary for this study following example 6 in ASME V&V40–2018.

Context of use	Model influence	Decision consequence	Model risk
Patient-specific FE model used to assess the subsidence risk of an implant (Table 3)	MEDIUM	LOW	LOW

The ASME V&V40 standard suggests several credibility factors that should be evaluated to demonstrate that the overall credibility of the computational model agrees with that of the model risk (Table 8).

A mesh convergence study of the bone structures was performed by calculating changes in the maximum FFR for gradually decreasing element edge length. Mesh convergence of the implant structures was assessed by decreasing the element edge length of the implant mesh while maintaining an element edge length of 1.5 mm for the bone structures. The maximum FFRs were calculated, as well as the percentage difference in the maximum FFR between different meshes ( $\frac{\left(current-previous\right)}{previous}*100$ ). The simulations were run using patient 2 and the ACD in titanium. Furthermore, for the mesh convergence study, a homogeneous Young’s modulus of 350 MPa for the bone structures was used.

The sensitivity analysis of the maximum FFR with respect to the load magnitude was performed by calculating the variation in the maximum FFR while applying variations (2.5% and 25% change), positive or negative, to the applied load magnitude. The sensitivity analysis of the maximum FFR to different apparent density-to-modulus relationships was performed by calculating the maximum FFR using two alternative relationships, from the study by Kopperdahl and Keaveny (1998) and by Morgan et al. (2003), with all other model parameters kept the same (Table 5). The sensitivity of the maximum FFR with respect to the constitutive model for the subsidence risk assessment was investigated by additionally calculating the maximum FFR with a linear constitutive model and a symmetric post-yield behaviour for the bone structures with all other model inputs equal. The sensitivity studies were conducted for patient 2 with the ACD in titanium and the AMCD of patient 6 in PEEK. Percentage differences were calculated compared to the base model, and the maximum percentage differences were reported. In our previous study, mechanical tests were conducted, and the computer model was subsequently validated against them (Smit et al., 2024) (Smit, 2023)*.* A more in-depth explanation of the credibility factors is provided in the work by Viceconti et al. (2016) and Aldieri et al. (2023).

**TABLE 5:** Apparent density-to-Young’s modulus relationships used in sensitivity study with Ouyang et al. as baseline.

Density-to-Young’s modulus relationships	
Ouyang et al. (1997) (baseline)	$E=2383{\rho_{app}}^{1.88}$
Kopperdahl and Keaveny (1998)	$E=2100\rho_{app}-80$
Morgan et al. (2003)	$E=4730{\rho_{app}}^{1.56}$

### 2.6 Statistical analysis

For statistical analysis, Spearman’s rank correlation coefficient analysis was used to calculate the correlation coefficient *r* with *(−1 < r < 1)* between two variables. A Mann–Whitney *U* test was used to compare two samples with statistical significance assumed when *p* < 0.05. The small sample size in this study does not allow for reliable normality testing (Taylor, 2017); therefore, statistical methods were selected that allow for non-normally distributed data and are robust on small datasets, e.g*.*, the median was reported. SciPy version 1.11.1 (Virtanen et al., 2020) and Seaborn version 0.11.2 (Waskom, 2021) were used to implement the statistical methods and data visualisations.

## 3 Results

### 3.1 Optimised cages

The resulting AMCDs show a “box-like” design and are hollow in the inside with patient-specific internal strut structures, similar as found by Smit et al. (2024) and (Smit, 2023). The outside shape conforms to the endplate shapes of the vertebrae. The designs satisfy all the constraints and reached a discreteness parameter lower than 3%. The morphological details of the AMCDs are listed in Table 6. The porosity and mean pore size diameter are very similar for the titanium and PEEK implants, the reason being that the local volume constraint, which controls the porosity and pore size distribution, is an active constraint. Nevertheless, the internal structures and the exact location of the pores are patient-specific. The fact that the local volume constraint is effective in controlling the implant morphology, independent of other constraints, enables it to tune the porous internal structure of the implant to be favourable for bone in-growth.

**TABLE 6:** Mean +/− standard deviation of porosity and pore diameter for AMCDs.

	Titanium implant	PEEK implant
Porosity	58.47% ± 1.1%	59.44% ± 1.5%
Mean pore diameter	5.01 mm ± 0.54 mm	4.88 mm ± 0.43 mm

### 3.2 Subsidence risk assessment

**FIGURE 4:**
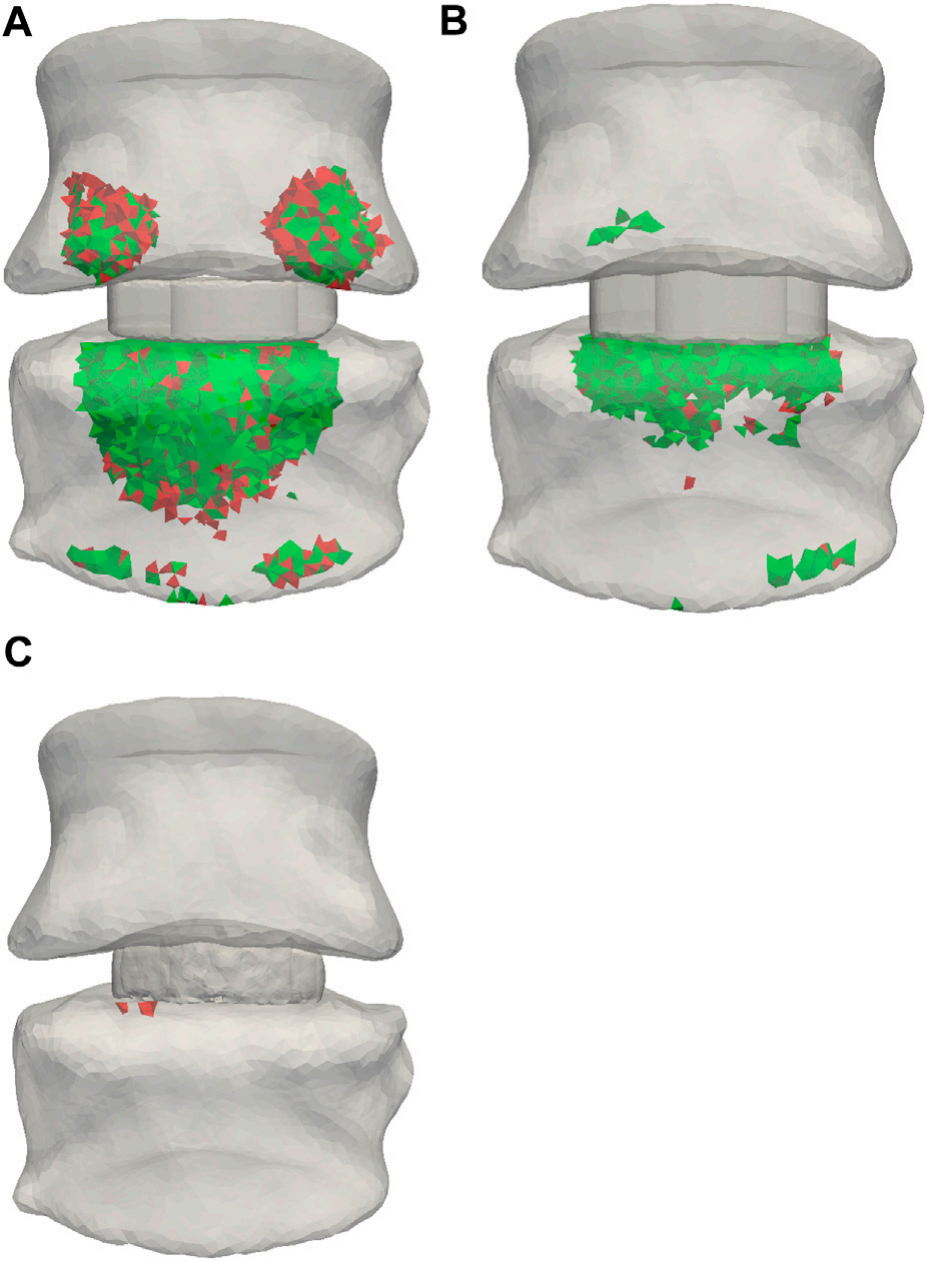
Elements with FFR >1 are highlighted with green for PEEK implant material and red for titanium implant material. **(A)** OTS model. **(B)** ACD model. **(C)** AMCD model. Data from patient 1.

Figure 4 highlights elements with FFR larger than 1, which indicates mechanical overloading, for patient 1 for all three implant types. The red elements are associated with the titanium implant and the green elements with the PEEK implant. Figure 4 shows that the titanium implant leads to a higher volume of overloaded elements than the PEEK implant material by 15%, 5%, and 300%, for the OTS cage, ACD, and AMCD, respectively. Furthermore, the two separate concentrations in the superior vertebra compared to the evenly distributed overloaded elements in the lower vertebra are explained by the contact areas between the endplate and the OTS cage (Figure 4A). The ACD and AMCD were endplate conforming and show that the regions of overloaded elements were reduced (Figures 4B, C).

**FIGURE 5:**
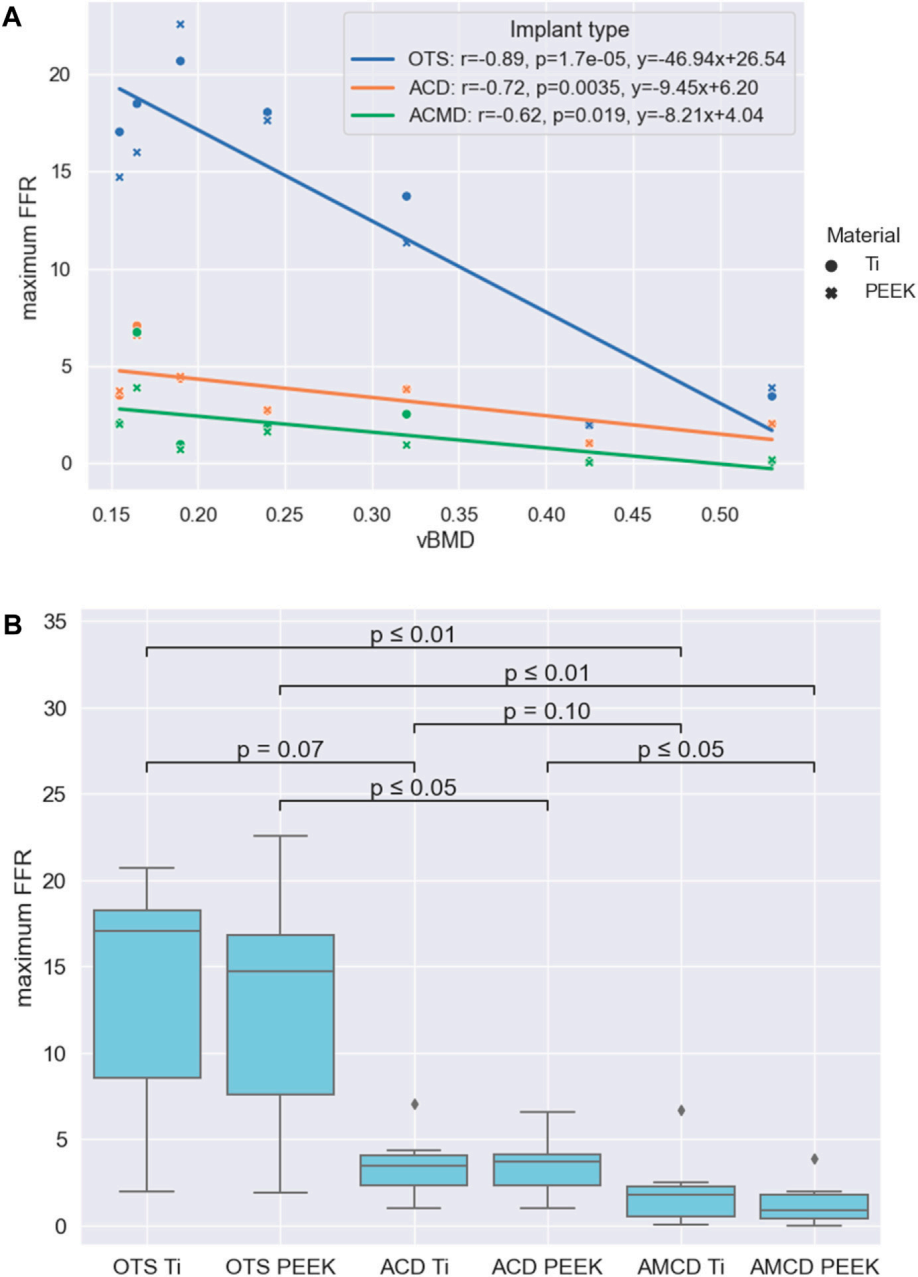
Results summarised: **(A)** Relationships between the maximum FFR and vBMD for the three implant groups. **(B)** Statistical significance of the reduction between different groups formed by the implant type and implant material.

The subsidence risk assessment provided the maximum FFR data for the three implant groups in PEEK and titanium implant materials for all patients (Figure 5A). In general, the subsidence risk for ACDs was reduced, compared to the OTS cages, and the subsidence risk for AMCDs was reduced, compared to the ACD, for all patients.

Comparison of the maximum FFR results between PEEK and titanium implant materials, for the three implant types, showed that there was no statistical difference between the PEEK and titanium groups with *p*-values of 0.71, 0.95, and 0.53 for the OTS, ACD and AMCD implant types, respectively. The negative correlations between the maximum FFR and vBMD for the different implant types are given in Figure 5A. As an illustration, the linear correlations between the maximum FFR and vBMD indicate that, for a patient with a mean vBMD of 0.25 g/cm^3^, the maximum FFR values would be 14.8, 3.8, and 2.0 for the OTS cage, ACD, and AMCD, respectively.

A comparison of the implant type and implant materials is given in Figure 5B. This shows that the median maximum FFR reduction between the OTS PEEK to AMCD PEEK, OTS Ti to AMCD Ti, and ACD PEEK to AMCD PEEK is statistically significant with 94%, 89%, and 75%, respectively. The reduction in the maximum FFR for the OTS cage to the ACD, ACD to AMCD, and OTS cage to AMCD is not strongly significantly correlated with vBMD, with *p*-values of 0.18, 0.07, and 0.09 for the PEEK implant material and 0.12, 0.15, and 0.09 for titanium, respectively.

### 3.3 Computer model credibility assessment

**FIGURE 6:**
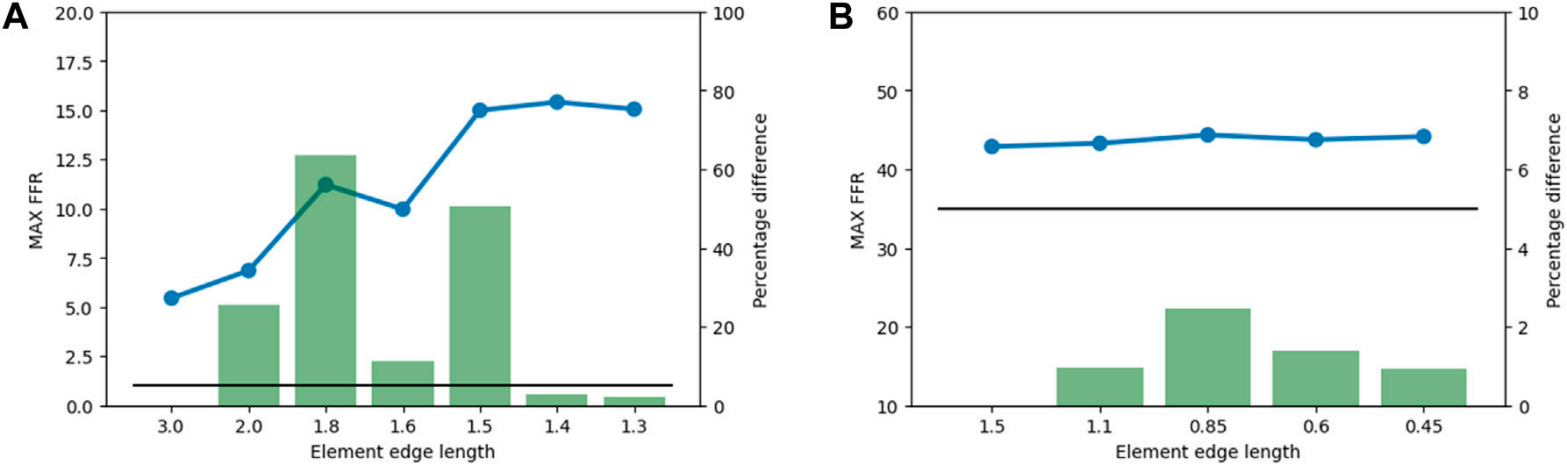
Results from the mesh convergence study. The maximum FFR value is indicated by the blue line, the percentage difference is indicated by the green bars, and the 5% threshold is indicated by the black line. **(A)** Mesh convergence results for bone structures in the subsidence risk assessment. **(B)** Mesh convergence results of implant structures in the subsidence risk assessment.

An element edge length of 1.5 mm was chosen for the bone structures because subsequent decreases in element edge length produced percentage differences in the maximum FFR lower than the 5% threshold (Figure 6A). For the implant structures, an element edge length of 0.5 mm was chosen, and the mesh convergence study indicated that variations had little influence on the maximum FFR (Figure 6B).

Table 7 shows the results of the sensitivity studies. The sensitivity of the maximum FFR with respect to variations in load magnitude, different density-to-Young’s modulus relationships, and different constitutive models is illustrated by reporting the maximum percentage difference derivation from the base case. Patient 2 is loaded in the linear loading regime and shows little variation in the maximum FFR for the applied variations. On the other hand, patient 6 shows a larger variation in the maximum FFR because this patient is loaded in the non-linear loading regime.

**TABLE 7:** Overview of the sensitivity study. The maximum percentage difference deviation from the base case is reported. Variation load magnitude: sensitivity of the maximum FFR with respect to the variation in the load magnitude. Relationships: sensitivity of the maximum FFR regarding different density-to-Young’s modulus relationships. Constitutive model: sensitivity of the maximum FFR with respect to the constitutive model.

Patient	2	6
Implant type	ACD	AMCD
Material	Titanium	PEEK
Variation load magnitude with 2.5%	0.2%	17.9%
Variation load magnitude with 25%	1.5%	76.5%
Density-to-Young’s modulus relationships	−0.8%	−0.1%
Constitutive models	0.0%	108%

The credibility factors were assessed after the outputs of the verification and validation (V&V) activities were obtained. The robustness of the activities and level of rigour were evaluated according to the guidelines and examples from the ASME V&V40–2018 standard. This assessment is summarised in columns “Rigour selected” and “Achieved credibility” in Table 8.

**TABLE 8:** Outcome of credibility assessment. Overview of the selected credibility activities according to The American Society of Mechanical (2018) in the first column. The number of the corresponding paragraph numbers within the ASME V&V40–2018 standard is reported in brackets. The classification in the “Rigour selected” column comes from the ASME V&V Standard. The last column refers to the credibility level that is achieved.

Activity categories		Credibility factor	Rigour selected	Achieved credibility
Verification	Code (5.1.1)	Referring to Dassault’s quality system	b	Medium
	Calculation (5.1.2.1)	Mesh convergence of the bone domain	b	Medium
		Mesh convergence of the implant domain	b	Medium
		Sensitivity of the maximum FFR to magnitude of load	b	Medium
Validation	Computational model (5.2.1.2.1)	Sensitivity of the maximum FFR of different density relationships	b	Medium
		Sensitivity of the maximum FFR to constitutive model	b	Medium
	Comparator (5.2.2.1 + 5.2.2.2)	Bench test validation was provided by Smit et al. (2024) and Smit (2023)	b-c-b-b-b-a-a-a-b	Medium
	Assessment (5.2.3)	Assessment of the rigour and achieved credibility	b-b-b-c-c	High
Applicability	Relevance to the quantity of interest (5.3.1)	Section 3.3	a	Low
	Relevance to CoU (5.3.2)	Section 3.3	b	Medium

The quantities of interest considered in the verification and validation study were the maximum FFR and the force–displacement curve in the mechanical tests. Therefore, we consider the quantities of interest relevant but not identical since the maximum FFR cannot be calculated directly in mechanical testing of samples. In the validation study, the range of applied forces is physiological and, therefore, deemed relevant for the CoU. The mechanical tests did not include the bone structures; therefore, partial overlap between the validation and CoU is concluded.

## 4 Discussion

The aim of this study was to perform *in silico* medical device testing of AMCDs, which were designed and optimised by our recently developed full-scale TO strategy, and compare the subsidence risk to OTS cages and ACDs. We performed the study on seven patient datasets using titanium and PEEK implant materials. We hypothesised that AMCDs would reduce subsidence risk over OTS cages and ACDs. We found that AMCDs reduce the subsidence risk compared to OTS cages and ACDs and that the median reduction is statistically significant for OTS PEEK compared to AMCD PEEK, OTS Ti to AMCD Ti, and ACD PEEK to AMCD PEEK (Figure 5B). Similar results were published by Chatham et al*.,* who compared a custom-shaped cage with a standard cage and found a significant reduction in stresses at the bone–implant interface (Chatham et al., 2017); however, they did not explore mechanically conforming devices.

Furthermore, we hypothesised that PEEK cages would have lower subsidence risk than titanium cages; however, our data did not show this to be the case. Other research studies confirm this observation and conclude that the implant geometry has a larger influence on subsidence risk than the implant material (Ferguson et al., 2006; Chatham et al., 2017; Suh et al., 2017). However, Carpenter et al*.* concluded in a computational study that PEEK interbody cages produce strain states in the adjacent vertebra that favour bone in-growth compared to titanium cages (Carpenter et al., 2018).

Lastly, we hypothesised that there is a negative correlation between subsidence risk, represented by the maximum FFR, and bone quality. We found that this negative correlation exists and is statistically significant for all three cage types (Figure 5A). This finding is in line with that of the previous literature (Jost et al., 1998; Schreiber et al., 2014; Tempel et al., 2015; Soliman et al., 2022). For example, Tempel et al*.* concluded that the rate of subsidence following lateral lumbar interbody fusion (LLIF) is negatively related to BMD, and patients diagnosed with osteopenia (DXA T-score of −1.0 or less) are at an increased risk of subsidence and revision surgery. Soliman et al*.* showed a higher vertebral bone quality score (a bone quality score derived from magnetic resonance imaging) that was significantly associated with an increased risk of subsidence.

We investigated how effective the optimisation method is in reducing the subsidence risk for patients with different bone densities; however, no significant correlation was found between the reduction for the maximum FFR and vBMD, suggesting that the effectiveness of the method is not strongly related to the bone quality of the patient. A possible explanation is the phenomena of sclerosis or thickening of the bony endplate in combination with degenerative disc disease (Velnar and Gradisnik, 2023), (Fields et al., 2018). Polikeit et al*.* reported that a thickening of the endplate can shift the load to the endplate, away from the vertebral body, reducing the influence of vertebral vBMD (Polikeit et al., 2003).

Performing credibility activities enhances the understanding of the behaviour of the computer model to assess the subsidence risk. First, looking at the results for patient 2, the influence of the applied variations had only a minor influence on the maximum FFR (Table 7). This is important to know because the load that is applied per patient in the present study is indirectly selected through the weight of the patient and from a small dataset, and therefore, small variations are likely compared to the *in vivo* population-wide situation. The risk exists that the loads are underestimated and lead to a significantly different AMCD design, resulting in an underperforming implant. However, the risk of implant failure is low since titanium implants are strong compared to the loads they are subjected to *in vivo*. Looking at patient 6, we observe a larger variation in the maximum FFR. The reason is that this patient has relatively low bone quality. Therefore, patient 6 is loaded in the non-linear loading regime, explaining the variability. The constitutive model variation is shown to result in a small influence when the model is operated in the linear regime. When the model is operated in the non-linear regime, we see a large influence on the maximum FFR, as is the case for patient 6. This confirms the choice for a non-linear material model for the subsidence risk assessment. The variations in the maximum FFR by changing the density-to-Young’s modulus relationships are small for both patients 2 and 6, and the relationship that is used in this study is the worst case.

As *in silico* trials are increasingly used in research, the authors found it useful to include extensive V&V activities according to the ASME V&V40–2018 standard (The American Society of Mechanical Engineers ASME, 2018). Although the standard was followed as much as possible in our assessment of model credibility, a certain degree of interpretation is involved in selecting the credibility activities and judging the rigour and the achieved credibility. We established the credibility of the model through an assessment that involved evaluating the relevance of validation activities and results in connection with the quantities of interest and predefined CoU (Tables 3, 8). Based on a review of the CoU, the model risk, the credibility activities, and results, the computer model for subsidence risk assessments is deemed sufficiently credible for the CoU. Compared to the credibility assessment by Aldieri et al. (2023), this study included less extensive mesh convergence and sensitivities studies but a similar degree of validation using mechanical test data with the exception of the use of human cadaveric material. This corresponds to the difference in the defined model risk, medium *versus* low for this study. Viceconti et al. (2021) specifically pointed out that a newly developed *in silico* computer model might be deemed credible for a CoU but has minimal clinical applicability because the clinical environment, where the computer model is intended to be used, is misunderstood. Therefore, they recommend that authors, reviewers, and editors should, at least, include the basic activities from the categories, namely, verification, validation, and uncertainty quantification, in any publication that includes mechanistic modelling.

A limitation in this study was the use of continuum modelling and the surrogate measure, the maximum FFR, as a representation of subsidence risk. Using continuum models, in the worst case, could result in sub-optimal cage designs; however, several studies did show that continuum models can accurately represent the mechanical response of the trabecular structures (Verhulp et al., 2006; Eswaran et al., 2009; Enns-Bray et al., 2018). Additionally, the vertebra endplates distribute the loads that are applied on the cage and reduce the influence of the continuum modelling assumption. The FFR is a measure based on principal strains that is valid across a range of bone densities (trabecular and cortical bone). Several authors have adopted and validated principal strain-based fracture criteria (Soyka et al., 2016) (Schileo et al., 2008) (Helgason et al., 2009). On the other hand, stress-based fracture criteria are often adopted in Molinari et al. (2021). Subsidence is a predictor of revision surgery (Tempel et al., 2018) (Campbell et al., 2020); however, the accuracy of the maximum FFR to predict subsidence and/or revision surgery should be investigated in future research, e.g*.*, in a retrospective study. A retrospective study can be performed with the combination of pre-operative CT scans, post-operative radiographic information on implant placement, and post-operative subsidence information. Longitudinal information would provide a more direct link between the maximum FFR and clinical outcomes. A second limitation was that the credibility activities according to the ASME V&V40–2018 standard likely need to be extended when the subsidence risk assessment would be used in a regulatory filing by including additional code verification activities, e.g., sensitivity studies on solver parameters, and comparator validations. Furthermore, the effect of cage height on the design was not studied separately and should be addressed in future work. A third limitation pertains to the stability or stiffness of the bone–implant system, which is not specifically quantified in this study. The stiffness of the bone–implant system is important since achieving stability or stiffness of the bone–implant system is the primary goal of a fusion surgery. A final limitation is the small sample size and the single source of the CT scan data. In the future, a larger sample size should be included to obtain a better understanding of the effectiveness of the method.

## 5 Conclusion

In this *in silico* medical device testing study, we demonstrated that anatomically and mechanically conforming devices achieved a median reduction in subsidence risk by 89% for titanium and 94% for PEEK, compared to off-the-shelf implants. Comparing an anatomically and mechanically conforming cage to an anatomically conforming cage, a median reduction of 75% is achieved for PEEK implant material through additional mechanical optimisation. We could not show a significant dependency between the achieved reduction and bone quality. A credibility assessment of the *in silico* medical device testing procedure to assess subsidence risk was performed according to ASME V&V40–2018, and the subsidence risk assessment was deemed sufficiently credible for the context of use.

## Data Availability

The datasets presented in this study can be found in online repositories. The names of the repository/repositories and accession number(s) can be found at: https://github.com/thsmit/TopOpt_in_PETSc_wrapped_in_Python.

## References

[B1] Action A. Viceconti M. Henney A. Morley-Fletcher E. (2016). In-silico clinical trials: how computer simulation will transform the biomedical industry. 10.13140/RG.2.1.2756.6164

[B2] Aldieri A. Curreli C. Szyszko J. A. La Mattina A. A. Viceconti M. (2023). Credibility assessment of computational models according to ASME V&V40: application to the bologna biomechanical computed Tomography solution. Comput. Methods Programs Biomed. 240, 107727. 10.1016/j.cmpb.2023.107727 37523955

[B3] Au A. G. Aiyangar A. K. Anderson P. A. Ploeg H. L. (2011). Replicating interbody device subsidence with lumbar vertebrae surrogates. Proc. Inst. Mech. Eng. Part H. J. Eng. Med. 225 (10), 972–985. 10.1177/0954411911415198 22204119

[B4] Bayraktar H. H. Morgan E. F. Niebur G. L. Morris G. E. Wong E. K. Keaveny T. M. (2004). Comparison of the elastic and yield properties of human femoral trabecular and cortical bone tissue. J. Biomech. 37 (1), 27–35. 10.1016/S0021-9290(03)00257-4 14672565

[B5] Bergmann G. Damm P. (2008). Orthoload. Berlin: Julius Wolff Institute, Berlin Institute of Health at Charité Universitätsmedizin Berlin. Accessed February 1, 2009.

[B6] Cadman J. Sutterlin C. III Dabirrahmani D. Appleyard R. (2016). The importance of loading the periphery of the vertebral endplate. J. Spine Surg. 2 (3), 178–184. 10.21037/jss.2016.09.08 27757430 PMC5067271

[B7] Campbell P. G. Cavanaugh D. A. Nunley P. Utter P. A. Kerr E. Wadhwa R. (2020). PEEK versus titanium cages in lateral lumbar interbody fusion: a comparative analysis of subsidence. Neurosurg. Focus 49 (3), E10–E19. 10.3171/2020.6.FOCUS20367 32871573

[B8] Carpenter R. D. Klosterhoff B. S. Torstrick F. B. Foley K. T. Burkus J. K. Lee C. S. (2018). Effect of porous orthopaedic implant material and structure on load sharing with simulated bone ingrowth: a finite element analysis comparing titanium and PEEK,” J. Mech. Behav. Biomed. Mater., 80, 68–76. 10.1016/j.jmbbm.2018.01.017 29414477 PMC7603939

[B9] Chatham L. S. Patel V. V. Yakacki C. M. Dana Carpenter R. (2017). Interbody spacer material properties and design conformity for reducing subsidence during lumbar interbody fusion. J. Biomech. Eng. 139 (5), 0510051–0510058. 10.1115/1.4036312 28334320 PMC5446564

[B10] EMA, “Guideline on the reporting of physiologically based pharmacokinetic (PBPK) modelling and simulation,” vol. pp. 3–15. 2018, Available at: https://www.ema.europa.eu/en/documents/scientific-guideline/adopted-reflection-paper-use-extrapolation-development-medicines-paediatrics-revision-1_en.pdf.

[B11] Enns-Bray W. S. Bahaloo H. Fleps I. Ariza O. Gilchrist S. Widmer R. (2018). Material mapping strategy to improve the predicted response of the proximal femur to a sideways fall impact,” J. Mech. Behav. Biomed. Mater., 78, 196–205. 10.1016/j.jmbbm.2017.10.033 29172124

[B12] Eswaran S. K. Fields A. J. Nagarathnam P. Keaveny T. M. (2009). Multi-scale modeling of the human vertebral body: comparison of micro-CT based highresolution and continuum-level models. Pac. Symp. Biocomput. 303, 293–303. 10.1142/9789812836939_0028 19209709

[B13] Ferguson S. J. Visser J. M. A. Polikeit A. (2006). The long-term mechanical integrity of non-reinforced PEEK-OPTIMA polymer for demanding spinal applications: experimental and finite-element analysis. Eur. Spine J. 15 (2), 149–156. 10.1007/s00586-005-0915-5 15940477 PMC3489413

[B14] Fields A. J. Ballatori A. Liebenberg E. C. Lotz J. C. (2018). Contribution of the endplates to disc degeneration. Curr. Mol. Biol. Rep. 4 (4), 151–160. 10.1007/s40610-018-0105-y 30546999 PMC6287619

[B15] Han K. S. Rohlmann A. Zander T. Taylor W. R. (2013). Lumbar spinal loads vary with body height and weight. Med. Eng. Phys. 35 (7), 969–977. 10.1016/j.medengphy.2012.09.009 23040051

[B16] Helgason B. Pálsson H. Rúnarsson T. P. Frossard L. Viceconti M. (2009). Risk of failure during gait for direct skeletal attachment of a femoral prosthesis: a finite element study. Med. Eng. Phys. 31 (5), 595–600. 10.1016/j.medengphy.2008.11.015 19150253

[B17] Jost B. Cripton P. A. Lund T. Oxland T. R. Lippuner K. Jaeger P. (1998). Compressive strength of interbody cages in the lumbar spine: the effect of cage shape, posterior instrumentation and bone density. Eur. Spine J. 7 (2), 132–141. 10.1007/s005860050043 9629937 PMC3611229

[B18] Kaiser J. Allaire B. Fein P. M. Lu D. Adams A. Kiel D. P. (2020). Heterogeneity and spatial distribution of intravertebral trabecular bone mineral density in the lumbar spine is associated with prevalent vertebral fracture. J. Bone Min. Res. 35 (4), 641–648. 10.1002/jbmr.3946 PMC7145746 31886907

[B19] Kassab-Bachi A. Ravikumar N. Wilcox R. K. Frangi A. F. Taylor Z. A. (2023). Contribution of shape features to intradiscal pressure and facets contact pressure in L4/L5 FSUs: an in-silico study. Ann. Biomed. Eng. 51 (1), 174–188. 10.1007/s10439-022-03072-2 36104641 PMC9831962

[B20] Kopperdahl D. L. Keaveny T. M. (1998). Yield strain behavior of trabecular bone. J. Biomech. 31 (7), 601–608. 10.1016/S0021-9290(98)00057-8 9796682

[B21] La Mattina A. A. Baruffaldi F. Taylor M. Viceconti M. (2023). Statistical properties of a virtual cohort for *in silico* trials generated with a statistical anatomy atlas. Ann. Biomed. Eng. 51 (1), 117–124. 10.1007/s10439-022-03050-8 36066781 PMC9832093

[B22] Lee D. C. Hoffmann P. F. Kopperdahl D. L. Keaveny T. M. (2017). Phantomless calibration of CT scans for measurement of BMD and bone strength—inter-operator reanalysis precision. Bone 103, 325–333. 10.1016/j.bone.2017.07.029 28778598 PMC5636218

[B23] Lowekamp B. C. Chen D. T. Ibáñez L. Blezek D. (2013). The design of simpleITK. Front. Neuroinform. 7, 45–14. 10.3389/fninf.2013.00045 24416015 PMC3874546

[B24] Molinari L. Falcinelli C. Gizzi A. Di Martino A. (2021). Effect of pedicle screw angles on the fracture risk of the human vertebra: a patient-specific computational model. J. Mech. Behav. Biomed. Mater. 116, 104359. 10.1016/j.jmbbm.2021.104359 33548583

[B25] Morgan E. F. Bayraktar H. H. Keaveny T. M. (2003). Trabecular bone modulus-density relationships depend on anatomic site. J. Biomech. 36 (7), 897–904. 10.1016/S0021-9290(03)00071-X 12757797

[B26] Niinomi M. Nakai M. (2011). Titanium-based biomaterials for preventing stress shielding between implant devices and bone. Int. J. Biomater. 2011, 1–10. 10.1155/2011/836587 PMC3132537 21765831

[B27] Nikiel P. Wróbel M. Szczepanik S. Stępień M. Wierzbanowski K. Baczmański A. (2021). Microstructure and mechanical properties of Titanium grade 23 produced by selective laser melting. Arch. Civ. Mech. Eng. 21 (4), 152–216. 10.1007/s43452-021-00304-5

[B28] Ouyang J. Yang G. T. Wu W. Z. Zhu Q. A. Zhong S. Z. (1997). Biomechanical characteristics of human trabecular bone. Clin. Biomech. 12 (7–8), 522–524. 10.1016/S0268-0033(97)00035-1 11415763

[B29] Passini E. Britton O. J. Lu H. R. Rohrbacher J. Hermans A. N. Gallacher D. J. (2017). Human *in silico* drug trials demonstrate higher accuracy than animal models in predicting clinical pro-arrhythmic cardiotoxicity. Front. Physiol. 8, 1–15. 10.3389/fphys.2017.00668 28955244 PMC5601077

[B30] Pauchard Y. Fitze T. Browarnik D. Eskandari A. Pauchard I. Enns-Bray W. (2016). Interactive graph-cut segmentation for fast creation of finite element models from clinical ct data for hip fracture prediction. Comput. Methods Biomech. Biomed. Engin. 19 (16), 1693–1703. 10.1080/10255842.2016.1181173 27161828 PMC5871234

[B31] Polikeit A. Ferguson S. J. Nolte L. P. Orr T. E. (2003). The importance of the endplate for interbody cages in the lumbar spine. Eur. Spine J. 12 (6), 556–561. 10.1007/s00586-003-0556-5 12783287 PMC3467986

[B32] Rohlmann A. Graichen F. Bender A. Kayser R. Bergmann G. (2008). Loads on a telemeterized vertebral body replacement measured in three patients within the first postoperative month. Clin. Biomech. 23 (2), 147–158. 10.1016/j.clinbiomech.2007.09.011 17983694

[B33] Rohlmann A. Pohl D. Bender A. Graichen F. Dymke J. Schmidt H. (2014). Activities of everyday life with high spinal loads. PLoS One 9 (5), e98510–e98519. 10.1371/journal.pone.0098510 24866883 PMC4035320

[B34] Rohlmann A. Zander T. Rao M. Bergmann G. (2009). Applying a follower load delivers realistic results for simulating standing. J. Biomech. 42 (10), 1520–1526. 10.1016/j.jbiomech.2009.03.048 19433325

[B35] Sarrami-Foroushani A. Lassila T. MacRaild M. Asquith J. Roes K. C. B. Byrne J. V. (2021). In-silico trial of intracranial flow diverters replicates and expands insights from conventional clinical trials. Nat. Commun. 12 (1), 3861–3912. 10.1038/s41467-021-23998-w 34162852 PMC8222326

[B36] Schileo E. Taddei F. Cristofolini L. Viceconti M. (2008). Subject-specific finite element models implementing a maximum principal strain criterion are able to estimate failure risk and fracture location on human femurs tested *in vitro* . J. Biomech. 41 (2), 356–367. 10.1016/j.jbiomech.2007.09.009 18022179

[B37] Schmitzer J. Strobel C. Blechschmidt R. Tappe A. Peuscher H. (2022). Efficient closed loop simulation of do-it-yourself artificial pancreas systems. J. Diabetes Sci. Technol. 16 (1), 61–69. 10.1177/19322968211032249 34328030 PMC8721541

[B38] Schreiber J. J. Hughes A. P. Taher F. Girardi F. P. (2014). An association can Be found between hounsfield units and success of lumbar spine fusion. HSS J. 10 (1), 25–29. 10.1007/s11420-013-9367-3 24482618 PMC3903949

[B39] Schroeder W. Martin K. Lorensen B. (1996). The visualization toolkit: an object oriented approach to 3D graphics. J. Aust. Entomol. Soc. 34, 335–342.

[B40] Smit T. (2023). Topology optimization of patient-specific spinal fusion implants. Switzerland: ETH Zurich.

[B41] Smit T. Aage N. Haschtmann D. Ferguson S. J. Helgason B. (2024). Anatomically and mechanically conforming patient-specific spinal fusion cages designed by full-scale topology optimization. J. Mech. Behav. Biomed. Mater. 159, 106695. 10.1016/j.jmbbm.2024.106695 39186906

[B42] Soliman M. A. R. Aguirre A. O. Kuo C. C. Ruggiero N. Azmy S. Khan A. (2022). Vertebral bone quality score independently predicts cage subsidence following transforaminal lumbar interbody fusion. Spine J. 22, 2017–2023. 10.1016/j.spinee.2022.08.002 35961523

[B43] Soyka R. P. W. Helgason B. Marangalou J. H. Van Den Bergh J. P. Van Rietbergen B. Ferguson S. J. (2016). The effectiveness of percutaneous vertebroplasty is determined by the patient-specific bone condition and the treatment strategy. PLoS One 11 (4), 1–14. 10.1371/journal.pone.0151680 PMC4839558 27100630

[B44] Suh P. B. Puttlitz C. Lewis C. Bal B. S. McGilvray K. (2017). The effect of cervical interbody cage morphology, material composition, and substrate density on cage subsidence. J. Am. Acad. Orthop. Surg. 25 (2), 160–168. 10.5435/JAAOS-D-16-00390 28009709

[B45] Taylor W. A. (2017). Statistical procedures for the medical device industry. Taylor Enterprises, Incorporated.

[B46] Tempel Z. J. Gandhoke G. S. Okonkwo D. O. Kanter A. S. (2015). Impaired bone mineral density as a predictor of graft subsidence following minimally invasive transpsoas lateral lumbar interbody fusion. Eur. Spine J. 24, 414–419. 10.1007/s00586-015-3844-y 25739988

[B47] Tempel Z. J. McDowell M. M. Panczykowski D. M. Gandhoke G. S. Hamilton D. K. Okonkwo D. O. (2018). Graft subsidence as a predictor of revision surgery following stand-alone lateral lumbar interbody fusion. J. Neurosurg. Spine 28 (1), 50–56. 10.3171/2017.5.SPINE16427 29125429

[B49] The American Society of Mechanical Engineers (ASME) (2018). Assessing credibility of computational modeling through verification and validation: application to medical devices, ASME V&V 40-2018. New York: ASME.

[B50] Velnar T. Gradisnik L. (2023). Endplate role in the degenerative disc disease: a brief review. World J. Clin. Cases 11 (1), 17–29. 10.12998/wjcc.v11.i1.17 36687189 PMC9846967

[B51] Verhulp E. van Rietbergen B. Huiskes R. (2006). Comparison of micro-level and continuum-level voxel models of the proximal femur. J. Biomech. 39 (16), 2951–2957. 10.1016/j.jbiomech.2005.10.027 16359680

[B52] Viceconti M. Henney A. Morley-Fletcher E. (2016). *In silico* clinical trials: how computer simulation will transform the biomedical industry. Int. J. Clin. Trials 3 (2), 37. 10.18203/2349-3259.ijct20161408

[B53] Viceconti M. Olsen S. Nolte L. P. Burton K. (2005). Extracting clinically relevant data from finite element simulations. Clin. Biomech. 20 (5), 451–454. 10.1016/j.clinbiomech.2005.01.010 15836931

[B54] Viceconti M. Pappalardo F. Rodriguez B. Horner M. Bischoff J. Musuamba Tshinanu F., (2021). “ *In silico* trials: verification, validation and uncertainty quantification of predictive models used in the regulatory evaluation of biomedical products,” Methods, 185, 120–127. 10.1016/j.ymeth.2020.01.011 31991193 PMC7883933

[B55] Virtanen P. Gommers R. Oliphant T. E. Haberland M. Reddy T. Cournapeau D. (2020). SciPy 1.0: fundamental algorithms for scientific computing in Python. Nat. Methods 17 (3), 261–272. 10.1038/s41592-019-0686-2 32015543 PMC7056644

[B56] Wang F. Lazarov B. S. Sigmund O. (2011). On projection methods, convergence and robust formulations in topology optimization. Struct. Multidiscip. Optim. 43 (6), 767–784. 10.1007/s00158-010-0602-y

[B57] Waskom M. (2021). Seaborn: statistical data visualization. J. Open Source Softw. 6 (60), 3021. 10.21105/joss.03021

[B58] Wu J. Aage N. Westermann R. Sigmund O. (2018). Infill optimization for additive manufacturing-approaching bone-like porous structures. IEEE Trans. Vis. Comput. Graph. 24 (2), 1127–1140. 10.1109/TVCG.2017.2655523 28129160

[B59] Zhang M. Pu F. Xu L. Zhang L. Liang H. (2017). Development of an integrated CAD–FEA system for patient-specific design of spinal cages. Comput. Methods Biomech. Biomed. Engin. 20 (4), 355–364. 10.1080/10255842.2016.1233401 27626889

[B60] Zhao F. Li D. Jin Z. (2018). Preliminary investigation of poly-ether-ether-ketone based on fused deposition modeling for medical applications. Mater. (Basel) 11 (2), 288. 10.3390/ma11020288 PMC5848985 29439551

[B61] Zhao Y. Zhao K. Li Y. Chen F. (2020). Mechanical characterization of biocompatible PEEK by FDM. J. Manuf. Process. 56 (March), 28–42. 10.1016/j.jmapro.2020.04.063

